# Supplementary figures and images for: High‐dimensional multiomics reveals perturbations to IL‐6/IL‐6R axis and RUNX3 in CD4 + T cells during third‐trimester pregnancy
Source: Clin Transl Immunology. 2026 Apr 28;15(5):e70096. doi: 10.1002/cti2.70096 (PMC13125720; doi:10.1002/cti2.70096)

A

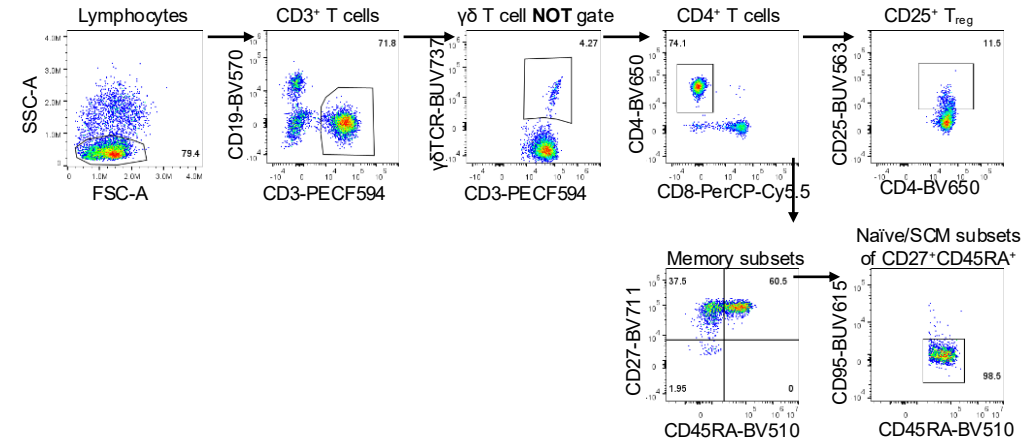

B

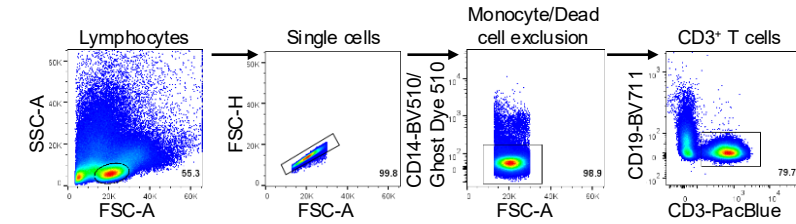

Habel et al Supplementary Figure 1

A

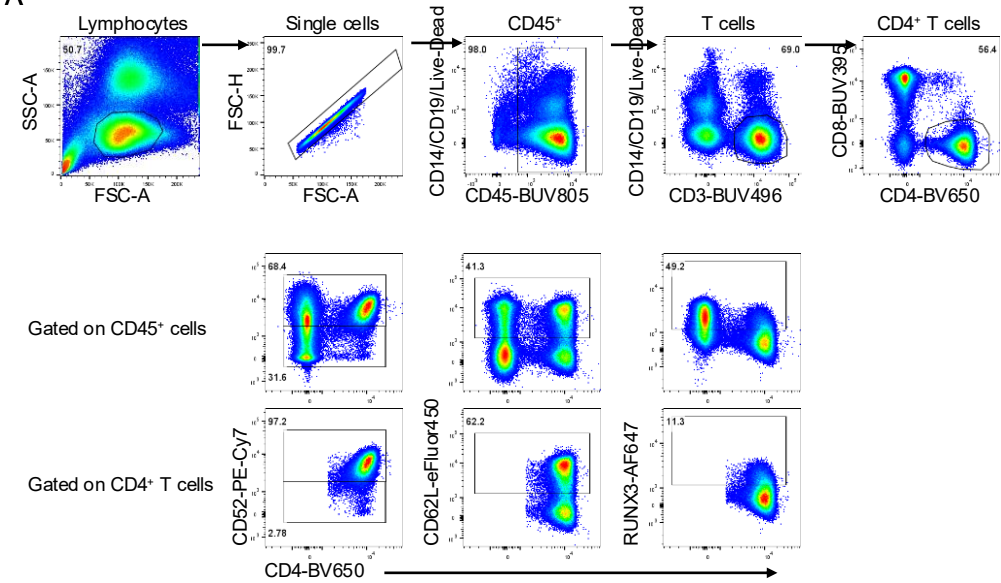

B

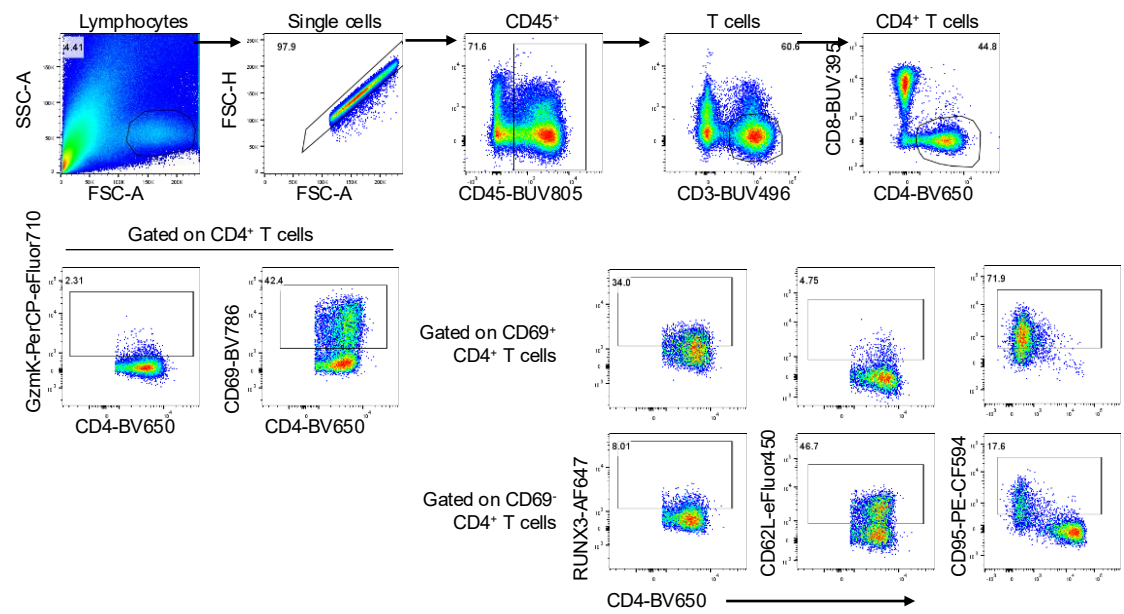

Habel et al Supplementary Figure 2

Supplement: Supplementary file 1 — Supplementary figure 1 Supplementary figure 2 [file CTI2-15-e70096-s001.pdf]
